# CHRONIC PAIN SELF-MANAGEMENT PRACTICES AND PREFERENCES AMONG URBAN AFRICAN AMERICAN OLDER ADULTS

**DOI:** 10.1093/geroni/igz038.273

**Published:** 2019-11-08

**Authors:** Mary R Janevic, Mary Janevic, Sheria Robinson-Lane, Susan Murphy, John Piette

**Affiliations:** 1 University of Michigan School of Public Health, Ann Arbor, Michigan, United States; 2 University of Michigan School of Nursing, Ann Arbor, Michigan, United States; 3 University of Michigan Medical School, Ann Arbor, Michigan, United States; 4 University of Michigan Sch Public Health, ann Arbor, Michigan, United States

## Abstract

African American older adults experience disproportionate burden from disabling chronic pain. Pain self-management interventions for this group are most effective when they integrate culturally-relevant preferences into intervention design. In the STEPS pilot trial, we collected focus group (n=23) and survey (n=57) data from African Americans age 60+ years about pain-management practices. Participants were recruited from the community and reported pain for 3+ months, with intensity >4 (0 to 10 scale). The most frequently-used pain-management strategies were exercise (75%) and prayer/Bible reading (74%). Also commonly used were healthy eating (61%), OTC medications (65%), and herbal supplements (51%). Focus group themes provided more nuanced information, including reasons for avoiding prescription pain medications, positive experiences with topical treatments, the value of movement, and the role of social support. Findings reveal strong engagement in pain self-care in this population. Interventions can build on existing practices by incorporating spirituality and appealing options for physical activity.

